# The effects of time-released garlic powder tablets on multifunctional cardiovascular risk in patients with coronary artery disease

**DOI:** 10.1186/1476-511X-9-119

**Published:** 2010-10-19

**Authors:** Igor A Sobenin, Valentin V Pryanishnikov, Lyudmila M Kunnova, Yevgeny A Rabinovich, Danik M Martirosyan, Alexander N Orekhov

**Affiliations:** 1 Institute of General Pathology and Pathophysiology, Russian Academy of Medical Sciences, Moscow 125315, Russia; 2 Moscow Municipal Cardiology Health Center, Moscow 121552, Russia; 3 Institute for Atherosclerosis Research, Moscow 121609, Russia; 4 Functional Food Center Inc., Richardson, TX 75080, USA

## Abstract

The double-blinded placebo-controlled randomized study has been performed in 51 coronary heart disease (CHD) patients to estimate the effects of time-released garlic powder tablets Allicor on the values of 10-year prognostic risk of acute myocardial infarction (fatal and non-fatal) and sudden death, with the respect of secondary CHD prevention. It has been demonstrated that 12-month treatment with Allicor results in the significant decrease of cardiovascular risk by 1.5-fold in men (p < 0.05), and by 1.3-fold in women. The above results were equitable also in terms of relative risks. The main effect that played a role in cardiovascular risk reduction was the decrease in LDL cholesterol by 32.9 mg/dl in men (p < 0.05), and by 27.3 mg/dl in women. Thus, the most significant effects were observed in men, while in women the decrease of cardiovascular risk appeared as a trend that might be due presumably to the insufficient sample size. Since Allicor is the remedy of natural origin, it is safe with the respect to adverse effects and allows even perpetual administration that may be crucial for the secondary prevention of atherosclerotic diseases in CHD patients.

## Background

Coronary heart disease is the most common cause of mortality. Risk factors for cardiovascular disorders include: hyperlipidemia, hypertension, obesity, and glucose intolerance. The social, medical and economical burden of CHD and the other complications of advanced atherosclerosis continue to increase in spite of the evident drop in cardiovascular death rates in the United States of America and several European countries. This fact is mostly due to the population ageing, the rapid increase in the prevalence of type 2 diabetes mellitus and obesity. These unfavorable tendencies are characteristic for many industrial and developing countries, therefore CHD remains the major cause of mortality and morbidity in the world.

Many factors appear to contribute to the development of atherosclerosis including alterations in plasma lipids and lipoprotein levels, blood pressure regulation, platelet function and clotting factors, etc. In recent years special algorithms for the estimation of overall cardiovascular risk based on the results of major epidemiological studies have been developed, which allow for the complex impact of different risk factors [1]. On the other hand, current approaches to the prevention of atherosclerotic diseases usually employ the effect on isolated risk factors. However, the polyetiological nature of atherosclerosis allows for the suggestion that simultaneous complex reduction of several risk factors appears to be the most valid and clinically effective way for primary prevention of cardiovascular diseases. Several studies revealed that a reduction in blood pressure reduces the risk and incidence of cardiovascular diseases [2,3].

Garlic (Allium sativum L. fam. Alliaceae) is one of the best-researched herbal remedies. Traditionally, it has been employed to treat infections, rheumatism, heart disease, diabetes, and many other disorders. Experimentally, it has been shown to exert antihypertensive, antibacterial, and hypoglycemic actions [4]. Clinically, garlic has been evaluated for a number of conditions, including hypertension, hypercholesterolemia, diabetes, and for the prevention of arteriosclerosis and cancer [4]. Several studies have indicated that garlic and its constituents inhibit key enzymes involved in cholesterol and fatty acid synthesis [5-8]. However, the clinical evidence is far from compelling.

Garlic powder tablets, Allicor, were used as the drug capable of reducing different risk factors [9]. Garlic contains a number of biologically active compounds and is widely used as traditional medicine in many cultures. In recent years, antiatherosclerotic and cardiovascular-protective effects of garlic have been extensively evaluated. Evidence from numerous studies demonstrate that garlic-based preparations can bring about the normalization of plasma lipids, along with the enhancement of fibrinolytic activity, inhibition of platelet aggregation and reduction of blood pressure[10]. It has been also shown that garlic inhibits human squalene monooxygenase and HMG-CoA reductase, enzymes involved in cholesterol biosynthesis [11].

The present study was performed to evaluate the effectiveness of Allicor treatment in primary CHD prevention and its effects on the estimates of multifunctional cardiovascular risk. It was investigated the effects of time-released garlic powder tablets Allicor on the values of 10-year prognostic risk of acute myocardial infarction and sudden death, with the respect of secondary CHD prevention.

## Introduction

Clinical manifestations of atherosclerosis, mainly, CHD and myocardial infarction, remain the leading cause of mortality, morbidity, illness and disability in populations worldwide. Atherosclerosis is an important medical and social problem, so, the mechanisms of its development and progression are an important field of investigations. At present, a group of numerous clinical and biochemical conditions (risk factors) tightly associated with the development of atherosclerotic diseases is revealed on the basis of the results of prospective epidemiological studies. Usually, the strategy for prevention of cardiovascular diseases is aimed to the reduction of risk factors, and modern recommendations demand vigorous control of multiple risk factors, including regulation of lipid metabolism, blood pressure, coagulation and fibrinolysis systems and others. The polyetiological nature of atherosclerosis allows the suggesting that simultaneous complex reduction of several risk factors appears to be the most valid and clinically effective way to the prevention of cardiovascular diseases. In recent years the algorithm for the assessment of multifunctional cardiovascular risk was developed from the data of PROCAM Study, that is suitable for use in CHD patients for the prediction of such cardiovascular events as myocardial infarction and sudden death. This model of risk assessment allows the estimation of the effects of different drugs that can modify risk factors on the dynamics of changes in cardiovascular risk that incredibly increases with ageing. Garlic-based preparations are thought to be promising agents for multifunctional risk reduction, since biologically active components of garlic possess a wide range of cardioprotective effects, such as lowering total and LDL cholesterol, increasing HDL cholesterol, lowering arterial blood pressure in mildly hypertensive subjects, enhancing fibrinolysis, and inhibiting platelet aggregation etc. [11,12]. However, the effectiveness of garlic-based drugs in secondary prevention of cardiovascular diseases is still obscure. The present double-blinded placebo-controlled randomized study has been performed to estimate the effects of time-released garlic powder tablets Allicor on the values of 10-year prognostic risk of acute myocardial infarction (fatal and non-fatal) and sudden death, with the respect of secondary CHD prevention in CHD patients.

## Patients and Methods

The study was performed in the department of primary prevention of cardiovascular diseases at Moscow Municipal Cardiology Health Center. Patients with documented CHD, men and women aged 40-65 years, who had a serum cholesterol level above 200 mg/dl (5.2 mmol/L) upon primary examination, were eligible for inclusion. The absence of high arterial hypertension (systolic blood pressure above 160 mm Hg or diastolic blood pressure above 95 mm Hg), or lipid-lowering drugs administration were also regarded as inclusion criteria. For randomization, patients were stratified according to gender, age, total cholesterol and smoking history. Two groups were formed, one who received Allicor (coated tablets containing 150 mg garlic powder, INAT-Farma, Moscow, Russia) one tablet twice a day for 12 months, and the other who received a placebo in the same manner. Allicor and placebo tablets looked identically.

Clinical and biochemical examination was performed upon the inclusion at the end of the study. Venous blood taken after overnight fasting was used for total cholesterol, triglycerides and high density lipoprotein (HDL) cholesterol measurements with commercial enzymatic kits (Boehringer Mannheim GmbH, Germany). Low density lipoprotein (LDL) cholesterol was calculated according to Friedewald formula.

Ten-year prognostic risk of fatal or non-fatal myocardial infarction and sudden death was calculated with Cox proportional hazards model derived from PROCAM study [13], where the following variables were used: gender, age, systolic blood pressure, total cholesterol, triglycerides, smoking status, diabetes mellitus, family history of acute myocardial infarction in 1^st ^degree relatives that occurred before the age of 60 years and after risk calculation, the regional adjustment factor was applied [14,15].

The significance of differences was estimated using SPSS 10.1.7 program package (SPSS Inc., USA). After the examination of variable distribution, the Mann-Whitney statistics or t-test was used for between-group comparisons, Wilcoxon statistics was used for within-group effect assessments, and Pearson's chi-square statistics was used for the comparison of nominal variables distributions. To estimate the relationship between changes in risk values and clinical and biochemical variables, Pearson's correlation analysis and regression analysis were used. The data is presented in terms of mean and S.E.M.; significance was defined at the 0.05 level of confidence.

## Results

Before the study, 92 patients were screened, and according to inclusion criteria 63 patients were recognized as eligible and randomized into two groups. Allicor-treated group consisted of 33 patients (18 men, 15 women), and placebo group consisted of 30 patients (17 men, 13 women). During the study, 7 patients (4 men, 3 women) dropped out of Allicor group, and 5 patients (3 men, 2 women) were lost in placebo group. The reason for the exclusion from the study was the discontinuation of study medication. So, the retirement accounted for 21.2% and 16.7% in Allicor-treated and placebo groups, respectively, and the difference between groups was not statistically significant. By the end of the study there were 26 evaluable patients in the Allicor-treated group (14 men, 12 women) and 25 in the placebo group (14 men, 11 women).

The baseline clinical data on evaluable patients are presented in Table 1. It can be seen that groups of patients did not differ significantly in all clinical variables, except the rate of smoking that was higher in Allicor recipients. In the placebo group, mean age was higher in women than in men (p < 0.05); with the respect to other variables, men and women did not differ significantly within groups.

**Table 1:** Clinical Characteristics of Patients at the Baseline.

	Allicor			Placebo		
	
Variable	All	Men	Women	All	Men	Women
	(n = 26)	(n = 14)	(n = 12)	(n = 25)	(n = 14)	(n = 11)
Age, years	56.7 ± 1.8	53.6 ± 1.9	60.3 ± 2.9	56.3 ± 1.7	52.6 ± 1.9	60.9 ± 2.4^$^
SBP, mm Hg	134.2 ± 2.8	130.4 ± 3.8	138.8 ± 3.9	130.8 ± 3.1	129.3 ± 4.4	132.7 ± 4.3
DBP, mm Hg	82.5 ± 1.9	82.1 ± 2.6	82.9 ± 2.9	81.0 ± 1.7	81.4 ± 2.5	80.5 ± 2.4
BMI, kg/m^2^	27.0 ± 0.9	27.2 ± 1.2	26.8 ± 1.5	27.9 ± 0.9	27.3 ± 1.0	28.6 ± 1.6
DM, n	0	0	0	0	0	0
LVH, n	3	2	1	3	2	1
MI, n	17	12	5	13	10	3
Smoking, n	8*	7	1	4	4	0

SBP, systolic blood pressure; DBP, diastolic blood pressure; BMI, body mass index, DM, type 2 diabetes mellitus; LVH, left ventricular hypertrophy; MI, myocardial infarction in anamnesis.

* - significant difference from placebo, p < 0.05;

^$ ^- significant difference between men and women within the group, p < 0.05.

Baseline lipid values are presented in Table 2. Allicor and placebo groups did not differ significantly in mean total cholesterol, HDL and LDL cholesterol and triglycerides levels. In placebo group, men and women differed significantly with the respect to HDL cholesterol (p < 0.05). In the Allicor-treated group, serum triglyceride levels were higher and HDL cholesterol was lower in men as compared to women, but the differences did not reach a statistical significance.

**Table 2:** The Changes in Serum Lipid Levels.

	Allicor			Placebo		
	
Time	All	Men	Women	All	Men	Women
	(n = 26)	(n = 14)	(n = 12)	(n = 25)	(n = 14)	(n = 11)
Total cholesterol, mg/dl						
At the baseline	269.2 ± 11.5	276.9 ± 16.3	260.2 ± 16.5	252.5 ± 9.1	259.4 ± 14.2	243.8 ± 10.4
After 12 months	235.7 ± 8.4*	237.0 ± 10.7*	234.1 ± 13.6	242.0 ± 6.9	238.9 ± 10.8*	245.8 ± 7.9
HDL cholesterol, mg/dl						
At the baseline	50.9 ± 3.6	45.3 ± 3.4	57.3 ± 6.4	48.7 ± 2.5	43.8 ± 1.8	54.9 ± 4.6^$^
After 12 months	51.5 ± 3.1	45.0 ± 2.4	59.0 ± 5.5^$^	50.7 ± 2.2	47.1 ± 2.2	55.2 ± 3.8
Triglycerides, mg/dl						
At the baseline	162.1 ± 19.5	182.1 ± 30.7	138.8 ± 22.0	127.8 ± 9.7	133.4 ± 12.9	120.6 ± 15.0
After 12 months	142.9 ± 16.5	148.6 ± 26.5	136.3 ± 19.2	106.9 ± 6.8*	108.6 ± 8.2*	104.8 ± 11.7
LDL cholesterol, mg/dl						
At the baseline	185.9 ± 9.3	195.2 ± 11.6	175.1 ± 14.8	178.3 ± 9.1	188.9 ± 13.5	164.8 ± 10.9
After 12 months	155.6 ± 7.8*^#^	162.3 ± 9.0*	147.8 ± 13.3	169.9 ± 7.2	170.1 ± 11.3*	169.6 ± 8.5

* - a significant difference from the baseline, paired Wilcoxon test, *P *< 0.05;

^# ^- a significant difference from placebo, Mann-Whitney test, *P *< 0.05;

^$ ^- a significant within-group difference between men and women, *t*-test, *P *< 0.05.

The changes in lipid levels that occurred during the study are shown in Table 2, as well. In the placebo group, no statistically significant changes were observed, except to triglycerides that lowered by 16.4% (95% CI: 5.1; 36.6 mg/dl, p = 0.004) from the baseline. However, in placebo-treated men the changes were rather prominent: total cholesterol decreased by 7.9% (95% CI: 3.7; 37.1 mg/dl, p = 0.020), LDL cholesterol decreased by 10.0% (95% CI: 1.3; 36.2 mg/dl, p = 0.048) and triglycerides decreased by 18.6% (95% CI: -1.1; 50.7 mg/dl, p = 0.030) from the baseline. On the opposite, in placebo-treated women only a slight insignificant decrease in serum triglycerides by 13.1% from the baseline was observed.

In Allicor-treated patients there were significant changes in total and LDL cholesterol levels. Total cholesterol decreased by 12.4% (95% CI: 12.3; 54.8 mg/dl, p = 0.004), and LDL cholesterol decreased by 16.3% (95% CI: 14.8; 45.8 mg/dl, p = 0.001) from the baseline. Cholesterol-lowering effects differed significantly from the placebo group (p = 0.038). Serum triglycerides lowered insignificantly by 11.8% from the baseline. Like in placebo group, the changes in lipid levels were more prominent in men than in women. So, the total cholesterol lowered by 14.4% (95% CI: 10.1; 69.7 mg/dl, p = 0.008), LDL cholesterol lowered by 16.9% (95% CI: 12.8; 53.0 mg/dl, p = 0.009) from the baseline, and statistically insignificant decrease in serum triglycerides by 18.4% was observed. In Allicor-treated women total cholesterol decreased by 10.0% (95% CI: -8.8; 61.0 mg/dl, p = 0.182) and LDL cholesterol lowered by 15.6% (95% CI: -0.4; 55.0 mg/dl, p = 0.060) from the baseline, but the changes did not reach a statistical significance possibly due to insufficient sample size.

The data on the changes in the 10-year prognostic risk of fatal and non-fatal myocardial infarction and sudden death is presented in Table 3. Since the was a substantial 8.5-fold difference in risk level between men and women, the analysis of changes between Allicor-treated and placebo groups without subdivision according to gender is inappropriate. In placebo-treated women, the risk of myocardial infarction and sudden death did not change significantly, and in placebo-treated men the risk lowered by 1.1-fold as compared to baseline (95% CI: -1,5%; 5,1%, p = 0.041). In Allicor-treated women the risk decreased by 1.3-fold (95% CI: -0.4%; 1.4%), but the difference did not reach statistical significance (p = 0.239). In Allicor-treated men the significant 1.5-fold decrease in risk level occurred (95% CI: -0.1%; 14.5%, p = 0.019).

**Table 3:** The Changes in 10-years Prognostic Risk of Myocardial Infarction and Sudden Death.

	Allicor			Placebo		
	
Time	All	Men	Women	All	Men	Women
	(n = 26)	(n = 14)	(n = 12)	(n = 25)	(n = 14)	(n = 11)
Absolute risk, %						
At the baseline	13.1 ± 3.8	22.3 ± 6.0	2.3 ± 0.6^$^	8.9 ± 2.4	14.3 ± 3.7	2.0 ± 0.7^$^
After 12 months	8.9 ± 2.3*	15.1 ± 3.5*	1.8 ± 0.6^$^	7.8 ± 2.4*	12.4 ± 3.9*	1.9 ± 0.6^$^
Relative risk						
At the baseline	1.00	1.00	1.00	1.00	1.00	1.00
After 12 months	0.81 ± 0.08*	0.76 ± 0.08*	0.88 ± 0.15	1.05 ± 0.13	0.81 ± 0.10	1.36 ± 0.23^$^

* - a significant difference from the baseline, paired Wilcoxon test, *P *< 0.05;

^$ ^- a significant within-group difference between men and women, *t*-test, *P *< 0.05.

The data on the changes in relative risk of myocardial infarction and sudden death is presented in Table 3, as well. Since relative values were used, it became possible to compare the between-group effects directly. In the placebo group relative risk tended to rise mainly due to the increase in age (95% CI: 0.79; 1.32), and in Allicor recipients relative risk decreased by 1.25-fold (95% CI: 0.64; 0.99, p = 0.019). Additionally, in Allicor-treated men the relative risk decreased by 1.3-fold (95% CI: 0.57; 0.94, p = 0.022). The main effect underlying the decrease in risk of myocardial infarction and sudden death in men was LDL cholesterol lowering (r = 0.409, p = 0.031 for absolute risk changes, and r = 0.463, p = 0.013 for relative risk changes).

## Discussion

The results of the given study have demonstrated that a 12-month treatment of CHD patients with garlic powder tablets Allicor results in the decrease of multifunctional prognostic risk of myocardial infarction and sudden death either prevents the rise of risk with ageing. The above effects were similar with the respect of absolute as well as relative risks. The most significant effects were observed in men, while in women the decrease of cardiovascular risk appeared as a tendency that might be due presumably to the insufficient sample size. It is notable that men differed from women significantly with the respect to a baseline prognostic risk level. This difference is based on the peculiarities of risk estimation algorithm, while in the PROCAM study only 32 myocardial infarctions occurred during the 10 year follow-up in 2,810 women aged 45-65, as compared to 325 events in 4,818 men in the same age group [13].

The results of major epidemiological studies have provided the evidence of cardiovascular risk determination by multifunctional effects of different risk factors, such as gender, age, family anamnesis, diabetes mellitus, dyslipidaemia, arterial hypertension, smoking history, and abdominal obesity, etc. [16]. The main approach in secondary CHD prevention is meaningfully based on the treatment of major risk factors. During the last decade, the conception of absolute multifunctional prognostic risk estimation that provides actual probability of acute cardiovascular event became prevailing in the planning of preventive therapy. The algorithms of multifunctional risk assessment gives better results even than the elevated LDL cholesterol level, the parameter that exceeds other isolated risk factors in terms of sensitivity and specificity [17]. The reason is that several risk factors can clusterize, thus increasing the probability of acute cardiovascular event in synergistic manner [18,19].

According to the polyetiological nature of atherosclerotic diseases, the key interventions in secondary CHD prevention include the correction of lipid metabolism, blood pressure control and smoking cessation. The lowering of blood cholesterol is traditionally regarded as the main method of CHD prevention. Indeed, the main effect of Allicor treatment that provided the decrease of multifunctional prognostic risk of myocardial infarction and sudden death in the given study was the significant decrease in total and LDL cholesterol. Lipid-lowering properties of garlic-based drugs and preparations are studied rather well [11,20,21], and in spite of the presence of contradictory results [18], the meta-analysis of performed clinical trials indicates that garlic treatment produces moderate hypolipidemic effect that may be quite comparable to rational dietary treatment [12,19]. Additionally, garlic-based preparation may produce moderate hypotensive effects in patients with mild or moderate arterial hypertension, and in some studies the increase of HDL cholesterol was demonstrated [10,22-26]. It is notable that garlic preparations influence significantly on platelet aggregation and plasma fibrinolytic activity that may alter the actual risk of myocardial infarction [16,21,27,28]. Although the last parameters are not taken in consideration in the algorithms of multifunctional risk estimation, they clearly act as independent and significant variables in the risk of cardiovascular events.

Garlic contains different biologically active components and sulfur-containing amino acids, such as allicin, ajoene, S-allylcysteine, S-methylcysteine, diallyl disulfide and sulfoxides, that may be responsible for antiatherosclerotic activity of garlic realized through different mechanisms of action [29,30]. Allicor contains garlic powder that is considered to retain the biologically active ingredients of raw garlic, both water-soluble and organic-soluble, in a better manner [31-33]. In contrast to other garlic-based drugs that are present at the market, Allicor possess a prolonged mode of action, since its biological effects last for 12-16 hours after single dose administration [34]. Since Allicor is the remedy of natural origin, it is safe with the respect to adverse effects and allows even the perpetual administration that may be crucial for the secondary prevention of atherosclerotic diseases in CHD patients.

## Conclusion

We have demonstrated that a 12-month treatment with Allicor results in the significant decrease of cardiovascular risk by 1.5-fold in men, and by 1.3-fold in women. The main effect that played a role in cardiovascular risk reduction was the decrease in LDL cholesterol by 32.9 mg/dl in men, and by 27.3 mg/dl in women. Thus, the most significant effects were observed in men, while in women the decrease of cardiovascular risk appeared as a trend that might be due presumably to the insufficient sample size.

## Abbreviations

(CHD): Coronary Heart Disease; (HDL): High Density Lipoprotein; (LDL): Low Density Lipoprotein

## Competing interests

The authors declare that they have no competing interests.

## Authors' contributions

IS participated in the design of the study and performed the statistical analysis. VP carried out the experimental studies. LK carried out the experimental studies. YR carried out the experimental studies. DM participated in the preparation of manuscript. AO participated in its design and coordination of the study. All authors read and approved the final manuscript.
